# Electroencephalography in Autism Spectrum Disorder

**DOI:** 10.3390/jcm14061882

**Published:** 2025-03-11

**Authors:** Magdalena Hankus, Patrycja Ochman-Pasierbek, Malwina Brzozowska, Pasquale Striano, Justyna Paprocka

**Affiliations:** 1 Department of Pediatric Neurology, Faculty of Medical Sciences, Medical University of Silesia, 40-752 Katowice, Poland; mhankus@sum.edu.pl; 2 Students’ Scientific Society, Department of Pediatric Neurology, Faculty of Medical Sciences, Medical University of Silesia, 40-752 Katowice, Poland; patrycja.ochman09@gmail.com (P.O.-P.); malwina.brzozowska@interia.pl (M.B.); 3 Pediatric Neurology and Muscular Diseases Unit, IRCCS Istituto Giannina Gaslini, Full Member of ERN-EPICARE, 16147 Genova, Italy; pstriano@unige.it; 4 Department of Neurosciences, Rehabilitation, Ophthalmology, Genetics, Maternal and Child Health, University of Genova, 16126 Genova, Italy

**Keywords:** EEG, autistic features, children, treatment, epilepsy

## Abstract

**Background/Objectives:** Electroencephalography (EEG) has been widely used to differentiate individuals with autism spectrum disorder (ASD) and co-occurring conditions, particularly epilepsy. However, the relationship between EEG abnormalities and core features of ASD remains unclear. This study reviews the potential impact of EEG findings on the development, behavior, sleep, and seizure occurrence in ASD patients. Additionally, it evaluates whether routine EEG testing is warranted for all ASD patients, particularly in the absence of clinical seizures. **Methods:** A systematic review was conducted that covered literature published between 2014 and 2024. The review focused on EEG abnormalities, both epileptiform and non-epileptiform, in individuals with ASD. Studies were selected based on predefined inclusion criteria, emphasizing the prevalence, type, and clinical relevance of EEG findings. The analysis also included a critical assessment of whether EEG abnormalities correlate with specific ASD symptoms, such as cognitive impairment, speech delay, or behavioral issues. **Results:** EEG abnormalities were reported in 23–80% of ASD patients, indicating a broad range of findings. Despite their frequent occurrence, the evidence linking these abnormalities to specific clinical symptoms remains inconclusive. Some studies suggest an association between epileptiform patterns and more severe ASD traits, while others do not confirm this. Furthermore, the effectiveness of anticonvulsant treatment in children with EEG abnormalities and no seizures remains uncertain, with limited supporting data. **Conclusions:** Given the uncertain relationship between EEG findings and ASD symptoms, routine EEG testing for all children with ASD appears unnecessary. EEG should be considered primarily when epilepsy is clinically suspected.

## 1. Introduction

Autism spectrum disorder (ASD) is a neurodevelopmental disorder characterized by impairments in social communication skills as well as restrictive repetitive behaviors, interests, or activities [1,2]. The prevalence of ASD has been steadily increasing over the past two decades, with current estimates reaching up to 1 in 36 children [3]. ASD is usually diagnosed using behavioral studies and observational scales, such as the Autism Diagnostic Observation Scale (ADOS), the Autism Screening Inventory for Infants and Toddlers (M-CHAT), and the Autism Diagnostic Interview Scale Revised (ADI-R) [4,5].

In recent years, methods like magnetic resonance imaging, functional magnetic resonance imaging, and electroencephalography (EEG), which can measure functional brain activity, have been employed to help analyze the differences between individuals with ASD and those with typical development [6,7,8,9].

In subjects with ASD, there is a wide range of cognitive skills; some individuals present with severe intellectual disability, and others with a high-level cognitive function. Additional neurological comorbidities like motor impairment, sleep disorders, and epilepsy are common and are associated with more clinical severity. Motor impairment manifests as delays in motor domains and deficits in praxis, coordination, and gait. The prevalence of epilepsy is estimated between 2% and 46%. Conversely, rates of ASD in epilepsy have been reported between 15% and 74% [10].

Across the literature, electroencephalography (EEG) could be helpful in differentiating individuals with ASD and some additional comorbidities. However, the relationship between epilepsy, epileptiform abnormalities without seizures, and core features of ASD is poorly understood. In some studies, epileptiform abnormalities on EEG without clinical seizures are considered part of the behavioral, language, and cognitive dysfunctions [11]. However, not all studies confirmed this thesis.

The aim of this study was to systematically review the available literature on EEG examinations in children with ASD to (1) assess the potential impact of EEG changes on the severity of autistic features, development, behavior, sleep, movements disorders, and occurrence of seizures and (2) answer the question whether there is an indication to routinely perform EEG examinations in all patients with autism? An additional aim was to investigate whether there are any implications for the treatment of children with ASD and EEG changes without a history of seizures.

### Key Points

- Routine EEG examinations in children with autism spectrum disorder (ASD) without a history of seizures often reveal both epileptiform and non-epileptiform abnormalities.
- A literature review on the relationship between observable EEG changes and the occurrence of disorders in several areas—such as the severity of autistic features, development, behavior, sleep, and movement disorders—does not allow for drawing definitive conclusions about the impact of these changes on the occurrence of the disorders.
- Some reports indicate that sodium valproate, levetiracetam, lamotrigine, and even corticosteroids have demonstrated efficacy in enhancing fundamental clinical functions; however, no studies that meet adequate statistical criteria have been conducted on this topic. There is a lack of precise data supporting the rationale for treating children with EEG changes in the absence of clinical seizures.
- Given the lack of clear evidence linking EEG findings to the progression of ASD and the absence of strong indications for treating EEG abnormalities without seizures, routine EEG testing in all children with autism appears unnecessary, except when epilepsy is suspected.

## 2. Methodology

### 2.1. Search Strategy

Our review focuses on original research assessing occurrence of EEG discharges in ASD patients and their potential influence on them. Searches on PubMed executed on 24 June 2024 using the research string “(eeg) OR (electroencephalo) AND (autis)” from 2014 to 2024 returned 1432 hits. The duplicates (7 articles) were subsequently deleted. Our research was conducted following the Preferred Reporting Items for Systematic Reviews and Meta-Analyses (PRISMA) guidelines.

### 2.2. Inclusion Criteria and Study Selection

**Figure 1:**
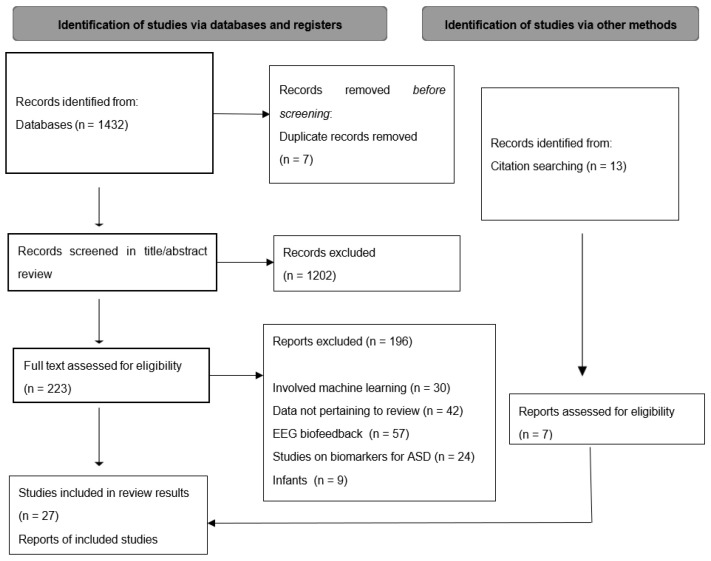
PRISMA flow diagram.

We included publications that were reviews, systematic reviews, or original studies, and that were available in English. Following a preliminary review of titles and abstracts, 223 articles were taken into consideration. The search process was also improved by reviewing reference lists for additional relevant articles. Ultimately, after a detailed analysis, we included 34 articles, 27 of which were published between 2014 and 2024. Due to the lack of newer evidence, we included an additional 7 articles regarding pharmacological treatment of patients with ASD and EEG abnormalities without seizures published before 2014. The exclusion criteria included studies on genetic mutations, Tuberous Sclerosis Complex, and those not focused on EEG (such as research on MEG, MRI, ERP, or SPECT). Studies not related to ASD (such as those on ADHD, Fragile X syndrome, or Smith–Magenis syndrome), as well as those involving machine learning, animal models, infants, EEG biofeedback, single case reports, and transcranial stimulation, were also excluded. The risk of bias across all studies and for individual studies was assessed. Disagreements were settled through discussion. The content of the final paper was thoroughly read, validated, and revised by all authors. A Prisma Flow Chart (Figure 1) has been incorporated to transparently depict the study selection process.

A formal risk of bias assessment was conducted using the Newcastle–Ottawa Scale (NOS) for observational studies and the Cochrane Risk of Bias Tool (RoB 2) for randomized controlled trials. The summarized findings are included in the Supplementary Materials.

## 3. Results

### 3.1. Type of EEG Abnormalities in ASD

The EEG findings in individuals without a history of seizures include epileptiform abnormalities (focal or generalized) and non-epileptiform abnormalities, such as slow activities or asymmetries in background rhythm. The frequency of a given type of discharge is variable. Considering the group of patients with ASD and abnormal EEG without seizures, epileptiform abnormalities could be found in 23–80% [12,13,14,15,16]. Additionally, Mulligan et al. showed that epileptiform abnormalities were rare in 56.4% of patients, recurrent in 27.3%, and frequent for 16.4% [16]. Several evidence suggest a notable prevalence of epileptiform abnormalities among individuals with regressive autism, reported at rates between 33% and 68% [17].

Epileptiform discharges could be found primarily during sleep. Santarone et al. showed that 95.5% of paroxysmal slowing and interictal epileptiform discharges were present only during sleep and in four cases (4.8%) also during wakefulness, specifically with a frequency 19.7 times higher during sleep [14]. In other studies, epileptiform abnormalities were observed in two-thirds during sleep only, whereas in the remaining one-third they were detected both during sleep and awake state [16,18]. However, in a minority (3.6%) the epileptiform abnormalities occurred in wakefulness only [16].

As far as the localization of epileptiforms is concerned, most of it is focal, but without predilection for specific locations. Capal et al. described focal epileptiform discharges in 83% of patients with abnormal EEG and most were seen in the left temporal region [12]. Mulligan et al. observed that the most frequent location was frontal, though all locations were represented, and many patients had multifocal or generalized epileptiform abnormalities [16]. Santarone et al. described focal abnormalities in 48.4% of recordings over central areas, 32.3% over temporal areas, and 19.3% over the frontal ones. However, focal discharges were seen only in one-third, whereas the remaining two-thirds were bilateral and diffuse [14].

Akhter et al. showed that EEG abnormalities were found in 51.9% of ASD participants (36.5% epileptiform, 15.4% non-epileptiform). Temporal and frontal regions had the most focal discharges (50% and 40%, respectively). A total of 89% of generalized abnormalities were symmetrical spike–wave complexes [19]. Other findings indicate that children with ASD showed reduced alpha frequency coherence in temporal-parietal and frontal regions on both sides compared to the controls [20].

In the study by Ronconi et al., EEG was used to investigate neural communication in the beta band. For the typical development group, there was a decrease in beta power following the stimulus onset, whereas the ASD group demonstrated an elevation in beta power linked to the event. Atypical oscillatory beta band activity (15–30 Hz) led to unusual functional connections between the occipital regions (which handle initial stimulus analysis) and the infero-temporal areas, responsible for extracting object identity [21].

Theta modulation impairments were correlated with autistic symptoms. In the research conducted by Larrain-Valenzuela et al. typically developing individuals exhibited parametric changes in alpha (positive fluctuation between 9 and 15 Hz) and theta band (elevation 5 to 8 Hz) power, but notable modulations were absent in ASD subjects [22]. Table 1. presents EEG alterations observed in patients with ASD.

### 3.2. EEG and Severity of Autistic Features

Some studies indicate a significant interaction between diagnosis and prevalence of epileptiform abnormalities. Mulligan et al. observed the lowest prevalence of epileptiform in patients with Asperger syndrome (20.0%) compared to 60.0% in autism and 81.3% in pervasive developmental disorders not otherwise specified [16]. Other studies also showed that sharp waves and other epileptiform were associated with more severe autistic features [13,17]. Nicotera et al. presented that only 7.14% of patients in the group without EEG abnormalities had a severe form of autism, whereas 38.8% of patients with epileptiform abnormalities showed severe autistic features (*p*  =  0.04) [18]. However, in some observations, no significant differences were noted [15].

### 3.3. EEG and Cognitive Skills

The issue is whether the epileptiform abnormalities found in children with ASD indicate an underlying cortical dysfunction that worsens clinical symptoms, or if these abnormalities merely coexist with the clinical manifestations, without a causal connection.

Nicotera et al. [18] showed an influence of EEG abnormalities on Intelligence Quotient (IQ). Most patients with EEG abnormalities had IQ deficits. All patients with severe IQ deficits showed EEG abnormalities (*p* = 0.04), whereas very few patients in the normal EEG group had moderate intellectual disabilities, and none had severe intellectual disabilities. A normal IQ was observed in 47.6% of subjects without EEG abnormalities. Similarly, Akhter et al. proved that epileptiform abnormalities were associated with moderate to severe intellectual disability (IQ < 50) [19]. Conversely, only 11.1% of patients with non-epileptiform abnormalities and 27.7% of those with epileptiform abnormalities had a normal IQ [18]. However, Santaroe et al. indicated that abnormal background activity during sleep is more associated with developmental delay than the presence of paroxysmal slowing and interictal epileptiform discharges [14].

The maturation of children’s brains is characterized by changes in electrical activity. Finn et al. measured this activity in both autistic and neurotypical children, identifying distinct developmental patterns in autistic children compared to their neurotypical counterparts. Peak alpha frequency (PAF) is identified as the alpha frequency with the highest absolute power, it delivers a more consistent representation of alpha frequency patterns. This is a sign of neural development that increases as children grow older. The elevation of PAF correlates with improvements in cognitive function. Findings from the study indicate that ASD children do not exhibit the typical age-dependent rise in PAF values. However, their PAF increases in relation to their cognitive skills [24].

### 3.4. EEG and Speech Development

Data on the impact of EEG changes on language development in children with ASD are inconsistent. In one study, most patients (66.6%) in the group without EEG abnormalities had mild verbal language impairment, while in the other, 33.33% had no language abilities at all. In stark contrast, 94.44% of patients in the epileptiform abnormalities group had no language abilities [18]. However, Mulligan et al. did not report any significant correlation between EEG abnormalities and language skills [16].

### 3.5. EEG and Behavioral Disorders

Research on behavioral disorders in children with ASD is difficult to compare due to the variety of characteristics considered. In some studies, no differences in EEG were found regarding social interaction, history of regression [12], emotional and behavior problems, hyperactivity, adaptative behavior [15], or anxiety level [18]. On the other hand, some studies showed a significant difference in the behavior problems between children exhibiting sharp waves and other waves [13] or more impaired adaptive functioning when compared with the group with normal EEG results [12]. Furthermore, the group with epileptiform abnormalities on their EEG had lower scores in executive functioning, particularly in inhibition control [15]. The study conducted by Neuhaus et al. focused on frontal alpha asymmetry (FAA) and their findings suggest that differences in FAA between individuals may be connected to mental health and social-emotional behaviors and that these connections may vary between males and females with ASD. For autistic males, FAA correlated with both social communication features and externalizing behaviors while for autistic females it correlated only with social communication features [23].

Nicotera et al. found that all patients with epileptiform abnormalities exhibited aggressive behaviors. In contrast, 85.7% of patients without EEG abnormalities did not display aggressive behaviors. There was also a significant positive correlation between epileptiform abnormalities and self-harm behavior [18].

### 3.6. EEG and Sleep

ASD is often accompanied by sleep disturbances. Arazi and co-authors investigated this issue. This study aimed to determine if slow wave activity (SWA), a marker of sleep pressure, is altered in children with ASD. Overnight EEG recordings from polysomnography evaluations of 23 typically developing children and 29 children with ASD were analyzed. The findings suggest a potential dysregulation of sleep homeostasis in ASD children, resulting in decreased sleep pressure. It was observed that children with ASD have lower SWA levels and shorter periods of slow-wave sleep compared to those without ASD. SWA power was also negatively correlated with their sleep onset times [25].

The analysis of delta EEG activity during NREM sleep also can yield substantial insights. This topic was explored by Rochette et al., who demonstrated atypical thalamo-cortical activity in the parieto-occipital area in individuals with ASD. An abnormal connection between the frontal cortex and sensorimotor memory encoding in patients with ASD was noted in their findings [26]. The issue of EEG changes during non-rapid eye movement sleep, which was also addressed by Lehoux et al. study on slow waves in NREM sleep that were suggested as a potential electrophysiological indicator of altered cortical maturation in ASD, associated with atypical thalamo-cortical network functioning [27].

### 3.7. EEG and Movements Disorders

Milovanovic et al. observed that epileptiform discharges in EEG in ASD patients correlated with lower motor skills scores on the Vineland Adaptive Behavior Scale II [28]. For motor stereotypies, 64.28% of patients showed the absence of EEG abnormalities. Rare motor stereotypies were evenly distributed across the EEG abnormality groups (55.5%). However, the proportion of subjects with noticeable and disruptive motor stereotypies was significantly higher in the epileptiform abnormalities group compared to the no-EEG abnormalities group (38.8% vs. 11.9%, respectively, *p*  <  0.01) [18].

The summary of the relationship between EEG changes and disorders in these six areas of development in children with ASD is presented in Table 2.

### 3.8. EEG and Epilepsy

A recent meta-analysis proved that nearly every fifth person with ASD suffers from coexisting epilepsy [29]. The frequency of epilepsy in ASD shows two peaks: early childhood and adolescence. However, another study suggests that the average prevalence of epilepsy in ASD doubles in adolescents [30]. Some studies indicate that there is a higher risk of autism with specific forms of epilepsy such as infantile epilepsy spasms syndrome (IESS) or Lennox–Gastaut syndrome [11]. Sharma et al. proved in a study on 100 children that the most common seizure types were generalized-onset tonic–clonic (48%), focal-onset with impaired awareness (17%), and focal to bilateral tonic–clonic seizures (17%) [31]. Shared neurobiological mechanisms have been found to likely be responsible for the co-occurrence of epilepsy, autism, and intellectual deficit [32]. Another possible explanation is the impact early life seizures may have on the function of neurotransmitter systems and intrinsic neuronal properties during neurodevelopment leading directly to disrupted cortical connectivity. The clinical expression of this derailment can result in seizures or devastating impairments in social communication and behavior, or both [30]. Studies showed that patients with coexisting ASD and epilepsy are the most frequently displayed interictal epileptiform discharges in the frontal lobe. Another frequent localization is the temporal lobe [30,33]. In a study conducted by Akhter et al. EEG abnormalities were found in 51.9% of ASD participants (36.5% epileptiform, 15.4% non-epileptiform). The epileptiform abnormalities were linked to epilepsy in 66.7% of participants [19]. What is more, it has been indicated that frontal paroxysms show a higher association with the development of epilepsy compared with centrotemporal paroxysm [33]. Patients with epilepsy alongside ASD showed higher rates of cognitive deficits and frontal lobe epileptiform activity compared with only ASD patients [30]. Children with ASD having co-occurring epilepsy conducted worse on developmental assessments and exhibited lower adaptive functioning than patients without epilepsy [12]. Intellectual disability has been found as an independent risk factor in the development of epilepsy in the setting of ASD [12]. The meta-analysis showed that for patients without intellectual disability, the rate of epilepsy was 8.9% compared with 23.7% in patients with intellectual disability [30].

### 3.9. Pharmacological Treatment of Patients with ASD and EEG Abnormalities Without Seizures

The issue of whether to treat seizure-free ASD patients who display EEG abnormalities remains a topic of scientific discussion. No studies that meet adequate statistical criteria have been conducted on this topic. Anticonvulsant efficacy is predominantly underscored by case study findings [33,34].

Reports indicate that sodium valproate, levetiracetam, lamotrigine, and even corticosteroids have demonstrated efficacy in stabilizing mood and enhancing fundamental clinical functions [33,34].

Wang et al. described the efficacy of levetiracetam (60 mg/kg/day) in reducing aggression and improving cognitive and behavioral functions in patients with ASD and EEG abnormalities without seizures, demonstrating a reduction in EEG discharges in 75% of cases [35]. Rugino et al. also reported that levetiracetam might play a role in mitigating aggression, hyperactivity, and impulsivity in children with autism spectrum disorder who display these behaviors [36].

According to the analysis of 176 patients treated with valproic acid (VPA) (doses from 80 to 120 mg/dL) with abnormal EEG results 46.6% (82 patients) showed subsequent normalization, 17.0% (30 patients) exhibited improvement without achieving normalization, 36.3% (64 patients) remained unchanged, and none deteriorated in the second overnight EEG. The clinical significance of this treatment for patients remains unknown, as no evaluation of clinical improvement has been conducted [37].

The findings of Hollander et al. suggested that divalproex sodium has the potential to mitigate EEG abnormalities, alleviate ASD symptoms, and improve social functioning. Caution is warranted in interpreting these results due to the retrospective nature of the study [38]. In a separate study, Hollander et al. highlighted the potential efficacy of divalproex sodium in managing repetitive and compulsive-like symptoms in patients with autism spectrum disorders (starting dose: 125 mg/day, increased by 125 mg every 4 days over 2 weeks) [39].

The research conducted by Pressler et al. on lamotrigine also warrants attention. Their findings revealed that children who had a decrease in EEG discharges during the lamotrigine phase also showed notable gains in their overall behavioral ratings [40].

However, Hirota et al. reported that antiepileptic drugs (valproate, lamotrigine, levetiracetam, and topiramate) were not more effective than the placebo [41] and the effectiveness of corticosteroids was ruled out by Duffy et al. A retrospective analysis identified 20 steroid-treated ASD patients and 24 untreated ASD patients. No substantial EEG differences were found over time, indicating EEG did not reflect treatment effects [42]. Additional double-blind, placebo-controlled trials reported no clinical progress in patients following treatment with levetiracetam [43] or lamotrigine [44].

Table 3 presents a summary of therapeutic approaches for patients with ASD accompanied by EEG abnormalities without seizures. Only studies that addressed EEG changes were included.

## 4. Discussion

Electrophysiological findings point to autism spectrum disorder being marked by irregularities in the anatomical and functional integration of neural circuits. While it is established that EEG patterns exhibit variance among individuals with autism, the precise characterization and clinical implications remain elusive. Applying EEG coherence to scrutinize electrophysiological connectivity patterns may delineate distinctions in neurological function between ASD and typically developing individuals [45].

The literature extensively documents atypical cerebral connectivity in autism spectrum disorders (ASD), but uncertainties persist regarding the precise nature of these anomalies and their functional relevance to distinct cortical rhythms. According to Shou et al., ASD is defined by divergent abnormalities (namely, reduced and increased connectivity) in the hierarchical structure of the entire brain [46]. Certain clinicians contend that regression in the autism spectrum of individuals constitutes an indication for conducting EEG assessments. The study by Kang et al. also validated the importance of regular EEG use. Their study analyzed 43 children aged 4 to 8 diagnosed with autism and contrasted their results with those of 43 typically developing children. The usefulness of EEG as a potential biomarker for assessing ASD risk in very young children has been suggested [47]. Dawson et al. similarly discussed this issue [48].

Counter-arguments against routine EEG monitoring have been put forward by Hughes et al. in 2015, suggesting that because of the high rate of non-significant anomalies, the limited number of positive diagnostic results, and the practical challenges in testing patients with ASD, the decision to implement this test should be made cautiously [49]. Even though we presented 10 years of research, the results are still inconclusive. A potential reason may be the small amount of available data and lack of meta-analysis. Additionally, autistic traits have a multifactorial basis, which could explain the discrepancies in EEG study results.

Another factor influencing the results could be the difficulty of conducting a sleep EEG study in children with more severe forms of autism. In a study conducted by Nicotera et al., 70 out of 139 patients were excluded because they did not undergo an EEG study in sleep and/or were not able to complete the neuropsychological protocol [18]. The implementation of mobile EEG technology for autistic children at home may increase access to research participation in this group. In the study, conducted by Giannadou et al., the feasibility of acquiring good-quality EEG data was demonstrated. The research included 69 children diagnosed with ASD, employing a gel-based EegoSports mobile system to collect EEG data [50].

One of the methods for enhancing the effectiveness of EEG application in ASD is the use of machine learning studies. Advancements in self-supervised learning (SSL) offer valuable insights into newer machine learning approaches that could enhance EEG analysis in ASD. SSL is especially advantageous given the challenges of obtaining large labeled EEG datasets, making it a promising strategy for improving the accuracy and reliability of EEG-based diagnostic tools in ASD research [51]. The use of neural network architectures for medical signal processing could also be adapted for EEG signal analysis in ASD. Feature preservation techniques might be especially useful for identifying subtle EEG patterns [52].

Neuroimaging analysis techniques could also be applied to EEG data processing in ASD. Multi-modal analysis approaches are increasingly important in understanding complex neurological conditions, ASD included [53]. Ali et al. proved the usefulness of the multi-stage convolutional neural network (CNN) framework to analyze MRI data, and the results presented provide insights into developing more precise EEG analysis techniques for ASD. The multi-stage framework approach could prove particularly relevant [54].

In the case of patients with autism and EEG changes without a history of seizures, the decision regarding treatment remains open-ended due to a lack of robust evidence for or against intervention. The analyzed studies often demonstrate a lack of statistical rigor. It is generally accepted that asymptomatic individuals should not undergo treatment. Nevertheless, some single studies suggest treatment should be considered in cases involving neurological symptoms or cognitive dysfunction [33,34]. Even though some patients with ASD do not experience seizures, EEG changes may suggest abnormalities in brain activity that can influence behavior and cognitive abilities. Recognizing these changes can lead to the implementation of therapies that support neurological development, potentially improving the social-emotional functioning of patients. In such situations, therapeutic options such as pharmacological treatment or behavioral therapy could be beneficial.

Nevertheless, further clinical research is required to determine whether targeted EEG therapy can lead to improvements in ASD symptoms. The observed EEG findings may be attributed to variations in patient characteristics, including medication status, age, and comorbid conditions. The impact of these factors as potential confounders should not be overlooked. Potential selection bias in patients with severe ASD should also be considered. Future research should ensure the selection of a representative sample.

Considerable heterogeneity in research methodologies, patient cohorts, EEG protocols, and outcome measures prevented a reliable meta-analysis from being performed.

## 5. Conclusions

Evidence is accumulating that some patients with ASD present abnormalities in EEG. However, given the ambiguous relationship between the EEG abnormalities and the clinical symptoms of autism spectrum disorder, it is not possible to draw definitive conclusions about the course of the condition based on this alone. At the same time, there is a lack of precise data supporting the rationale for treating children with EEG changes in the absence of clinical seizures. Therefore, routinely performing an EEG on all children with autism seems unnecessary, except for patients in whom epilepsy is suspected.

Further research and the establishment of standardized methods for assessing EEG discharges and their impact on clinical symptoms in ASD seem essential.

## Figures and Tables

**Table 1:** EEG changes observed in patients with autism spectrum disorder.

Study	EEG Abnormalities—Type/Specification	EEG Abnormalities—Localisation	Percentage of Patients with Abnormal EEG
Epileptiform Abnormalities			
Capal et al. [12]	-	83% * focal—most commonly left temporal 17% * diffuse	67.4%
Veerappan et al. [13]	sharp waves (33%) and other abnormal wave patterns (9%)	-	42%
Santarone et al. [14]	paroxysmal slowing and interictal epileptiform discharges (95.5% *—during sleep, 4.8% *—wakefulness and sleep)	37.7% * focal: (48.4% central 32.3% temporal 19.3% frontal) 62.7% * diffuse	28.4%
Romero-González et al. [15]	-	66.7% * focal (50% temporal-parietal 33.3% right temporal 16.7% central-temporal) 33.3% * diffuse	13%
Mulligan et al. [16]	56.4% rare 27.3% recurrent 16.4% frequent (3.6% * during wakefulness, 58.9% * during sleep, 37.5% *—wakefulness and sleep)	-	59.4%
Nicotera et al. [18]	spike, sharp waves, slow spike, and wave complexes (72% * during sleep, 27% *—wakefulness and sleep)	55.5% * focal (70% anterior areas 30% e posterior areas) 44.4% * diffuse (widespread anomalies and/or multifocal)	26.8%
Akhter et al. [19]	89% spike–wave complexes	33% * focal (50% temporal 40% frontal 7.4% multifocal sharp waves) 37% * diffuse	70.3% (36.5% of all participants)
Non-Epileptiform Abnormalities			
Capal et al. [12]	background slowing (most common)	47% focal slowing 65% generalized slowing	36.8%
Mulligan et al. [16]	slowing	-	21.8%
Nicotera et al. [18]	slowing and/or irregularity of the background rhythm	-	13.04%
Akhter et al. [19]	theta/delta slowing, excessive beta activity, or asymmetry	-	29.6% (15.4% of all participants)
Carson et al. [20]	alpha frequency interhemispheric coherence	-	-
Ronconi et al. [21]	atypical oscillatory beta band activity (15–30 Hz)	-	-
Larrain-Valenzuela et al. [22]	theta and alpha oscillation impairments	-	-
Neuhaus et al. [23]	frontal alpha asymmetry (FAA)	-	-

* percentage of patients with epileptiform abnormalities in EEG.

**Table 2:** Relationship of EEG changes with developmental disorders in children with autism spectrum disorder.

Development Area	Study	Results of the Study
Severity of autistic features	Veerappan et al. (2018) [13] Ghacibeh et al. (2015) [17]	Association between sharp waves and epileptiform dischargesand more severe autistic features.
	Romero-González et al. (2022) [15]	No significant differences.
	Mulligan et al. (2014) [16]	Frequency of epileptiform discharges: 20.0% patients with Asperger syndrome; 60.0% patients with autism; 81.3% patients with pervasive developmental disorder not otherwise specified.
	Nicotera et al. (2019) [18]	Severe form of autistic features: 7.14% in the group without EEG abnormalities; 38.8% in the group with EEG abnormalities.
Cognitive skills	Santaroe et al. (2023) [14]	Association between abnormal background activity during sleep and developmental delay.
	Nicotera et al. (2019) [18]	11.1% patients with non-epileptiform abnormalities had normal IQ. 27.7% patients with epileptiform abnormalities had normal IQ. No patient with normal EEG had severe intellectual disability.
	Akhter et al. (2021) [19]	64.7% of patients with moderate to severe intellectual disabilities had abnormal EEG. 72.2% of individuals with mild or no intellectual disabilities had normal EEG results.
	Finn et al. (2023) [24]	Children with ASD show atypical age-dependent rise in PAF values.
Speech development	Mulligan et al. (2014) [16]	No significant correlation between EEG abnormalities and language skills.
	Nicotera et al. (2019) [18]	Patients with normal EEG: 66.6% displayed a mild verbal language deficit; 33.33% showed no language. Patients with epileptiform abnormalities: 94.44% showed no language.
Behavioral disorders	Capal et al. (2018) [12]	No differences in EEG regarding social interaction and history of regression. ASD patients with EEG abnormalities show more impaired adaptive functioning than controls with normal EEG.
	Veerappan et al. (2018) [13]	Children with sharp waves had significantly more behavior problems compared to those with other waves.
	Romero-González et al. (2022) [15]	No differences in EEG regarding emotional and behavioral problems, hyperactivity and adaptive behavior. Worst performance on executive functioning in patients with epileptiform abnormalities.
	Nicotera et al. (2019) [18]	No differences in EEG regarding anxiety level. All patients with epileptiform abnormalities showed aggressive behaviors. Positive correlation between epileptiform abnormalities and self-harm behaviors.
	Neuhaus et al. (2023) [23]	Frontal alpha asymmetry (FAA): in autistic males correlated with social communication features and externalizing behaviors; in autistic females correlated only with social communication features.
Sleep	Arazi et al. (2020) [25]	ASD patients have lower slow-wave activity levels and shorter periods of slow-wave sleep.
	Rochette et al. (2018) [26]	Atypical thalamo-cortical activity in the parieto-occipital area during NREM sleep in children with ASD.
	Lehoux et al. (2018) [27]	Slow waves during NREM as a potential electrophysiological indicator of altered cortical maturation in ASD.
Movements disorders	Nicotera et al. (2019) [18]	Noticeable and disruptive motor stereotypies: 38.8% patients with epileptiform abnormalities; 11.9% patients with normal EEG.
	Milovanovic et al. (2021) [28]	Epileptiform discharges were associated with lower motor skill scores on the Vineland Adaptive Behavior Scale II.

**Table 3:** Summary of therapeutic approaches for patients with ASD accompanied by EEG abnormalities without seizures.

Drug/Method	Study	Description
Levetiracetam	Wang et al. (2017) [35]	Dose: 60 mg/kg/day The study demonstrated the efficacy of levetiracetam in reduction in EEG discharges in 75% cases while also improving behavioral and cognitive functions.
Valproic acid (VPA)	Chez et al. (2006) [37]	Dose: from 80 to 120 mg/dL A total of 46.6% of patients showed subsequent EEG normalization, 17.0% of patients exhibited improvement without achieving normalization, 36.3% of patients remained unchanged, and none deteriorated in the second overnight EEG.
Divalproex sodium	E. Hollander et al. (2001) [38]	Dose: 768 mg/day (average dose) Divalproex sodium has the potential to mitigate EEG abnormalities, alleviate ASD symptoms, and improve social functioning.
Lamotrigine	Pressler et al. (2005) [40]	Dose: 2 mg/kg/day (<12 yrs) or 150 mg/day (>12 yrs) for children with sodium valproate, 10 mg/kg/day (<12 yrs) or 300 mg/day (>12 yrs) without it Reduction in EEG discharges during the lamotrigine phase corresponded with a improvement in the global behavioral evaluation of the children.
Corticosteroids	Duffy et al. (2014) [42]	Dose: Oral prednisolone, 2 mg/kg/day There were no notable differences in EEG readings over time, indicating that the EEG did not reflect treatment effects.

## Supplementary Materials

The following supporting information can be downloaded at: https://www.mdpi.com/article/10.3390/jcm14061882/s1.
