# Supplementary material for: Electroencephalography in Autism Spectrum Disorder
Source: J Clin Med. 2025 Mar 11;14(6):1882. doi: 10.3390/jcm14061882 (PMC11943118; doi:10.3390/jcm14061882)
Supplement: Supplementary file 1 [file jcm-14-01882-s001.zip › jcm-3472949-supplementary.pdf]

Supplementary Materials

Table S1. Assessment of risk of bias for studies fulfilling the criteria of the Newcastle-Ottawa Scale (NOS).

Study	Selection (0-4)	Comparability (0-2)	Exposure/Outcome (0-3)	Total Score (0-9)	Risk of Bias
G.S. Bajestani, M. Behrooz and A.G. Khani et al.	*** 3	** 2	*** 3	7/9	Low
J.K. Capal et al.	**** 4	** 3	*** 3	5/9	Moderate
Veerappan et al.	*** 3	* 1	** 2	6/9	Moderate
Santarone et al.	*** 3	* 1	** 2	6/9	Moderate
M. Romero-González, P. Navas-Sánchez, E. Marín-Gámez et al.	**** 4	** 2	* 1	7/9	Low
Mulligan et al.	* 1	* 1	* 1	3/9	High
Nicotera et al.	** 2	* 1	* 1	4/9	Moderate
Akhter, S. et al	** 2	* 1	** 2	5/9	Moderate
Carson et al.	*** 3	* 1	** 2	6/9	Moderate
L. Ronconi et al.	**** 4	** 2	*** 3	9/9	Low
Neuhaus et al.	*** 3	** 2	** 2	7/9	Low
Arazi et al.	* 1	* 1	** 2	4/9	Moderate
Rochette et al.	*** 3	** 2	** 2	7/9	Low
Lehoux et al .	*** 3	** 2	*** 3	8/9	Low
Sharma et al.	*** 3	* 1	*** 3	7/9	Low

*cross-sectional study					
Chez et al.	*** 3	** 2	** 2	7/9	Low
Duffy et al.	*** 3	* 1	** 2	6/9	Moderate
Kang et al.	**** 4	* 1	** 2	7/9	Low
Hughes et al.	** 2	- 0	** 2	4/9	Moderate
Giannadou et al.	*** 3	* 1	** 2	6/9	Moderate

Table S2. Evaluation of the risk of bias for studies adhering to the criteria of the Cochrane Risk of Bias Tool for randomized controlled trials (RoB 2).

Study	Randomization	Allocation Concealment	Blinding	Missing Data	Outcome Reporting	Other Bias	Overall Risk of Bias
Larrain-Valenzuela J et al.	Low risk	Low risk	Some concerns	Low risk	Some concerns	Moderate	Moderate
Wang et al	Some concerns	Some concerns	Low risk	Low risk	Low risk	Some concerns	Some concerns
Hollander et al.	Some concerns	Some concerns	Low risk	Low risk	Low risk	Some concerns	Some concerns
Pressler et al.	Low risk	Low risk	Low risk	Some concerns	Low risk	Some concerns	Some concerns
Wasserman et al.	Some concerns	Some concerns	Low risk	Some concerns	Low risk	High risk	High risk
Belsito et al.	Some concerns	Some concerns	Low risk	Some concerns	Low risk	High risk	Some concerns
Shou et al.	High risk	High risk	Some concerns	Low risk	Low risk	Moderate	High risk
